# Dermatology: Where are We Coming from and Where are We Going to?

**DOI:** 10.3389/fmed.2014.00040

**Published:** 2014-10-24

**Authors:** Peter C. M. van de Kerkhof

**Affiliations:** ^1^Department of Dermatology, Radboud University Nijmegen Medical Centre, Nijmegen, Netherlands

**Keywords:** skin diseases, dermatology, dermatoses, targeted treatments, diagnosis

## Introduction

For centuries, skin diseases have been described according to their macromorphological appearance and classified according the morphological description. The system of grouping morphological features in skin diseases followed the same principles as defined by Carolus Linnaeus (1707–1778) in the taxonomy of plants and animals.

The major development of the morphological description of skin diseases was following extensive application of dermatopathology on skin biopsies. By integrating macro- and micromorphology, a new system for description of skin diseases had been created the primary efflorescences. The primary efflorescences constitute a set of macromorphological characteristics, which are indicative for the most important general pathological features (1). This system has served and still serves the classification of skin diseases. The primary efflorescences are the essentials in descriptions, diagnosis, and treatment of dermatoses. Dermatoses are classified from the point of view of the fundamental pathologic process involved. In other words, the classification is made according to the essential lesions.

During the last four decades important new insights in the genetics, pathophysiology, and cell biology have provided crucial information about the etiology and pathogenesis of skin diseases and have developed dermatology from a morphology driven discipline into an etiology based discipline. These observations have impacted the system of classification of skin diseases and the possibility for targeted treatments. The development of etiology based disease classification and targeted treatments have brought dermatology in better alignment with other medical disciplines such as internal medicine, rheumatology, and gastroenterology. In particular, the multidisciplinary approach in systemic diseases provides etiological concepts beyond the boundaries of the individual disciplines.

Skin diseases are easily accessible for inspection and investigation. That is why skin diseases are at the frontiers for advancement of insights in the etiology and pathogenesis of diseases and for understanding the mode of action of treatments.

A major question is whether classical dermatology in general practice can serve to the patients in the light of new developments? On one hand, there is a major challenge for teaching and continuing education. On the other hand, dermatology networks around centers of excellence are and will be of major importance in the future.

## Diagnosis of Skin Diseases

### Genodermatoses

Genetic technology over the past decade has generated new knowledge in the association of allelic variations in several genes with specific skin diseases. As of today, the genetic bases of the majority of the more common genodermatoses are known. Our insight into the biology of skin diseases has increased accordingly.

Genetic counseling has been greatly aided by gene identification, DNA-based prenatal diagnosis in several conditions, and DNA-based preimplantation diagnosis has been used. A summary of genodermatoses associated with allelic variations in known genes is provided in the classic review by Irvine and McLean (2).

Irvine and McLean defined important gaps in our knowledge and directions for future research:

- genotype–phenotype correlation: clinical heterogeneity implies that different phenotypes may arise from one mutation; genetic heterogeneity implies that the same phenotype may arise from different mutations
- variable penetrance may cause different phenotypes
- revertant mosaicism is a well-known phenomenon, which contribute to the complexity of phenotype – genotype correlations.

Further investigations of the underlying mechanisms will give better clues to the clinical understanding of the genetic abnormalities. For the nosology of skin diseases, classical morphological description will permit adequate diagnosis in many patients. However, a new approach is possible, focused on the genetic observations in stead of clinical descriptions (3–5). For clinical practice, new technology may provide new opportunities and also ethical challenges. In particular, with respect to prenatal diagnosis and postnatal diagnosis, the knowledge about a genotype may pose questions with respect to termination of pregnancies and questions to interventions intended.

## Inflammatory Dermatoses

Inflammatory dermatoses comprise a spectrum of different diseases. Based on the clinical description, different disease entities have been defined, which may be restricted to the skin or may be a systemic disease. Inflammatory diseases share or may have distinct pathogenetic processes, which may harbor a target for anti-inflammatory treatments.

For example, a disease such as psoriasis remain in the majority of patients restricted to a few erythematosquamous plaques, without any sign of systemic inflammation and can be treated with a topical treatment (vitamin D analog and/or corticosteroid). About 1/3 rd of patients with psoriasis has more widespread disease, with co-morbidities such as arthritis and metabolic syndrome. So far, the course of the disease is chronic and unpredictable.

The etiology of psoriasis is unknown. Several genes have shown to be involved (6). These genes comprise various pathogenetic processes such as IL-23 and IL 17 signaling, Interferon gamma signaling, NF-κB (nuclear factor kappa-light-chain-enhancer of activated B cells) signaling, epidermal proliferation, and keratinization. The question arises to what extent psoriasis harbors different genotypes, which are reflected in different phenotypes. Indeed, it has been shown that the chronic plaque psoriasis and pustular psoriasis are different diseases, with respect to genotype and phenotype (7). Further studies are needed to understand better, the differences in clinical aspects of psoriasis in the light of different genes involved in the individual patients.

Phototherapy, methotrexate, cyclosporin, acitretin, and fumarates are the most established classical treatments for psoriasis. In case of insufficient control treatment with anti-TNF biologics (etanercept, adalimumab, or infliximab) or with the anti Il12–23 (ustekinumab). It remains an art to select the appropriate treatment in an individual patient based on relevant co-morbidities and the requirement of therapeutic strength.

Every patient has his/her own psoriasis and that is true, in particular, with respect to treatment. Current interest is on development of co-diagnostics to predict the course of the disease and responsiveness to the various treatments. Genetic markers, immune markers, and pharmacological markers are helpful.

## Oncology

### Non-melanoma skin cancer

Non-melanoma skin cancer (NMSC) is the most frequent malignancies of the skin. During the last 3 decades an important increases of NMSC has been noticed, resulting from more extensive exposition of the skin to UV radiation. The diagnosis and subsequent treatment of premalignancies and malignancies belonging to NMSC are a major task for dermatologists and general practitioners. An important future possibility to facilitate rapid diagnosis is *in vivo* confocal microscopy (8). However, it has to be realized that the restricted *in vivo* penetration of the laser light is an important limitation of this method.

## Melanoma

Mutations in BRAF-oncogene are responsible for continuous growth in 60% of melanoma’s (9). Other mutations are in KIT – gene in acral, mucosal, and lentigo melanoma (10). Various kinase inhibitors inhibit kinases associated with continuous growth of melanoma via individual mutations. Further studies on relevant mutations and the molecular control of growth induced by these oncogenes may lead to a target therapy of advanced melanoma.

## Targeted Treatments

### Genodermatoses

Therapeutic strategies according to the underlying genetic mechanisms have been developed. The easy access to the skin compared with solid internal organs, provides unique opportunities for cutaneous gene therapy; however, many technical issues have to be addressed to enable gene replacement or modification of gene expression in genetic diseases.

With *ex vivo* cutaneous gene therapy, a gene therapy vector is incorporated in cultured keratinocytes derived from a patient s biopsy and then grafted back onto the patient’s skin using well-established technology developed for the treatment of burns and, more recently, for stable vitiligo (11). Mutant gene expression has been successfully rescued in cultured keratinocytes from a variety of recessive genodermatoses, including Recessive Dystrophic Epidermolysis Bullosa – Herlitz (RDEB-HS) (12) junctional epidermolysis bullosa, type Herlitz (JEB-H) (13), and others. The system has been used to treat successfully cells from patients with JEB-H (14) and X-linked ichthyosis (15).

*In vivo* gene therapy provides direct transfer of a therapeutic gene construct into the skin of the patient. One difficulty here is getting DNA past the highly evolved barrier function of the skin.

mRNA inactivation have been developed, based on short inhibitory RNA (siRNA) technology (16). Another RNA-based approach is that of RNA trans-splicing, aimed at correcting mutations in recessive or dominant conditions. This has recently been used to correct mutations in collagen XVII *in vitro* (17).

Gene repair oligonucleotides are small DNA–RNA hybrid molecules that can target and correct the mutation within a given gene.

## Inflammatory Dermatoses

Although many patients are treated with topicals, phototherapy, and laser treatments the major innovations were not primarily in this area. Development of pathogenesis based treatments was possible by the interventions with biologics targeted at specific sites in the pathogenesis of inflammatory diseases.

A variety of classical anti-inflammatory treatments and innovative targeted treatments (biologics and small molecules) are available. In particular, in the field of psoriasis new treatments are currently developed. These applications may often be of benefit for other inflammatory diseases.

Reverting to psoriasis, so far no early biomarkers, indicative for a more active course with more widespread disease or systemic involvement are available. So far, the question whether early active intervention is needed in at least some patients is not known (18).

During the course of the disease, it is very difficult to indicate with certainty, whether a systemic treatment with a classical antipsoriatic drug (methotrexate, fumarates, cyclosporin, or retinoids) is indicated in an individual, or whether the patient has to be treated with a biologic (either anti-TNF or ustekinumab).

So far, some evidence is available that plasma through levels of biologics are important to avoid under – and overdosing (19, 20) Some evidence is available that *TNFAIP3* gene polymorphism will be associated with better responsiveness to anti-TNF (21) and HLA-Cw6 but not LCE3B/3C deletion nor TNFAIP3 polymorphism will be associated with better responsiveness to ustekinumab (22).

In psoriasis, new treatments in development are the biologics with anti-IL-17 action, anti IL-23, and the small molecules apremilast (anti-phosphodiesterase 4) and tofacitinib (JANUS kinase inhibitor) (23–29).

Future research is focused on understanding, which treatment has to be given to which patient and when. Biomarkers and co-diagnostics to help guiding future treatment in the individual patients are of crucial value to provide optimal care.

With the development of small molecules, new innovative topical treatments will become reality in the near future. Pathogenesis based treatments will than innovate the management of frequently occurring more mild expressions of inflammatory skin diseases.

## Oncology

### Non-melanoma skin cancer

In addition to the traditional treatments such as surgery, cryotherapy and radiotherapy, and photo dynamic therapy, stimulation of cytotoxic immunity by imiquimod is an important advance (30). In future, further immunostimulatory treatments may provide important opportunities for targeted treatment of NMSC.

## Melanoma

Targeted therapy with inhibitors of the BRAF and MKE proteins is well-established for those patients with BRAF-mutant melanoma (31). However, following a clinical response, resistance develops in the tumors treated with these agents.

Another new approach is immunotherapy with antibodies, modulating the interaction between dendritic cells and T lymphocytes by blocking CTLA-4 and PD-1 (31–33). This results in augmented cell immunity and in a durable clinical response in melanoma patients.

## Future Prospects

Further studies beyond the boundaries of clinical classification may help to define stratification, which is relevant to the course of a disease and with respect to responsiveness to individual treatments. Indeed, personalized medicine will provide important future opportunities. This impacts our research, which so far often was focused on “group analyses,” into approach to focus on individual genotypical and phenotypical identifications. For the society, individualized personalized care will be more cost effective as unnecessary interventions can be restricted to focused individualized care.

## Conflict of Interest Statement

The author declares that the research was conducted in the absence of any commercial or financial relationships that could be construed as a potential conflict of interest.
